# Increased miR‐34c mediates synaptic deficits by targeting synaptotagmin 1 through ROS‐JNK‐p53 pathway in Alzheimer’s Disease

**DOI:** 10.1111/acel.13125

**Published:** 2020-02-24

**Authors:** Zhongli Shi, Kaixia Zhang, Huimin Zhou, Lei Jiang, Bing Xie, Ruiyuan Wang, Wenzhen Xia, Yajuan Yin, Zhaoyu Gao, Dongsheng Cui, Rui Zhang, Shunjiang Xu

**Affiliations:** ^1^ Central Laboratory The First Hospital of Hebei Medical University Shijiazhuang China; ^2^ Hebei International Joint Research Center for Brain Science Shijiazhuang China

**Keywords:** Alzheimer's disease, memory, miRNA, P53 protein, synaptic plasticity, synaptotagmin 1

## Abstract

Alzheimer's disease (AD) and cancer have inverse relationship in many aspects. Some tumor suppressors, including miR‐34c, are decreased in cancer but increased in AD. The upstream regulatory pathways and the downstream mechanisms of miR‐34c in AD remain to be investigated. The expression of miR‐34c was detected by RT–qPCR in oxidative stressed neurons, hippocampus of SAMP8 mice, or serum of patients with amnestic mild cognitive impairment (aMCI). Dual luciferase assay was performed to confirm the binding sites of miR‐34c in its target mRNA. The Morris water maze (MWM) was used to evaluate learning and memory in SAMP8 mice administrated with miR‐34c antagomir (AM34c). Golgi staining was used to evaluate the synaptic function and structure. The dramatically increased miR‐34c was mediated by ROS‐JNK‐p53 pathway and negatively regulated synaptotagmin 1 (SYT1) expression by targeting the 3′‐untranslated region (3′‐UTR) of syt1 in AD. The expression of SYT1 protein was reduced by over expression of miR‐34c in the HT‐22 cells and vice versa. Administration of AM34c by the third ventricle injection or intranasal delivery markedly increased the brain levels of SYT1 and ameliorated the cognitive function in SAMP8 mice. The serum miR‐34c was significantly increased in patients with aMCI and might be a predictive biomarker for diagnosis of aMCI. These results indicated that increased miR‐34c mediated synaptic and memory deficits by targeting SYT1 through ROS‐JNK‐p53 pathway and the miR‐34c/SYT1 pathway could be considered as a promising novel therapeutic target for patients with AD.

## Graphical abstract

miR‐34c mediated synaptic plasticity deficits by targeting SYT1 through ROS‐JNK‐p53 pathway in response to oxidative stress and participated in the pathogenesis of AD.

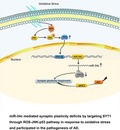

## 1 INTRODUCTION

Alzheimer's disease (AD), one of the most common neurodegenerative diseases, is characterized by progressive cognitive impairment clinically (Butterfield & Halliwell, 2019). β‐Amyloid (Aβ) plaques and neurofibrillary tangles are key neuropathological hallmarks in AD (Perez et al., 2019). Accumulation of Aβ peptide leads to synaptic loss (Zempel et al., 2013), neuronal death (He et al., 2019), and memory deficits (Yang et al., 2017), and the decrease in hippocampal synaptic plasticity is an early event in the pathogenesis of AD (Styr & Slutsky, 2018). Recently, some epidemiological researches have reported the negative association between AD and cancer, which means older persons with AD dementia have a reduced risk of cancer and vice versa (Calderwood & Murshid, 2017; Nixon, 2017; Shafi, 2016). Following studies have demonstrated that many factors, including miRNAs, are involved in the inverse relationship between AD and cancer (Nagaraj, Zoltowska, Laskowska‐Kaszub, & Wojda, 2019). Determining why AD and cancer seem to inhibit each other might open up important new areas of prevention and treatment research for both diseases.

MicroRNAs (miRNAs) are small, endogenous, noncoding RNAs that negatively regulate gene expression at the post‐transcriptional level by binding to the “seed region” located in the 3’‐untranslated region (3’‐UTR) of their target mRNA (Mahmoudi & Cairns, 2017). MiRNAs are considered as a group of regulators in various biological processes, such as cell proliferation, differentiation, apoptosis, and cycle regulation (Bhaskaran et al., 2019; Mansini et al., 2018; Nowakowski et al., 2018). Changes in miRNA expression may cause cellular malfunction and result in disease phenotypes, including AD and cancers (Nagaraj et al., 2019). To date, some miRNAs, including miR‐34c, have been identified to be dysregulated in the hippocampus, plasma, and cerebrospinal fluid of patients with AD (Muller, Kuiperij, Claassen, Kusters, & Verbeek, 2014). MiR‐34c is an important tumor suppressor and plays critical roles in many aspects of cancer biology, including invasion, metastasis, and angiogenesis (Piletic & Kunej, 2016). Dysregulated expression of miR‐34c is observed in some carcinomas, such as colorectal cancer (Gu, Wang, Liu, & Xiong, 2018), breast cancer (Liu et al., 2015), or even lung cancer (Kim et al., 2019). We have reported that miR‐34c was upregulated significantly during the progression of AD in the senescence‐accelerated mouse (SAMP8) model by miRNA microarray analysis (Zhou et al., 2016). The circulating levels of miR‐34c were increased in patients with AD (Bhatnagar et al., 2014), but decreased in patients with cancer (Zeng, Chen, Zhu, Luo, & Yang, 2017), compared to age‐matched normal controls, and inhibitor of miR‐34c ameliorates amyloid‐induced synaptic failure in mice (Hu et al., 2015). These findings indicate that miR‐34c, as a tumor suppressor, plays key roles in the pathological process of AD. However, the exact molecular mechanisms of miR‐34c and its upstream regulatory pathways in the development of AD have not yet been elucidated. Some functional miRNAs, including miR‐34 family, are differentially expressed in AD and cancer (Bhatnagar et al., 2014; Chamani, Sadeghizadeh, Masoumi, & Babashah, 2016; El Fatimy et al., 2018). We speculate that the tumor suppressor miR‐34c might play a role in the inverse relationship between AD and cancer.

The senescence‐accelerated mouse prone 8 (SAMP8) strain is a spontaneous animal model of accelerated aging developed by selective inbreeding of the AKR/J strain. As it spontaneously shows AD‐like cognitive and behavioral alterations including cognitive impairment, Aβ deposition, and tau hyperphosphorylation, it has been widely used as AD model to explore the etiopathogenesis of sporadic AD (Diaz‐Perdigon et al., 2019).

In this study, the expression patterns of miR‐34c were determined in oxidative stressed hippocampal neurons and SAMP8 mice. Then, the upstream signaling pathways and the downstream molecular mechanisms of increased miR‐34c were investigated in vitro and in vivo. Finally, the intervention effects of miR‐34c inhibitor were observed and the circulating miR‐34c levels were detected in patients with aMCI. The results of this study will provide evidence to elucidate the underlying mechanisms of AD pathogenesis and to further understand the relationship between AD and cancer.

## 2 RESULTS

### 2.1 Upregulation of miR‐34c, ROS generation, JNK activation, and P53 accumulation are involved in the pathological process of AD

**Figure 1:**
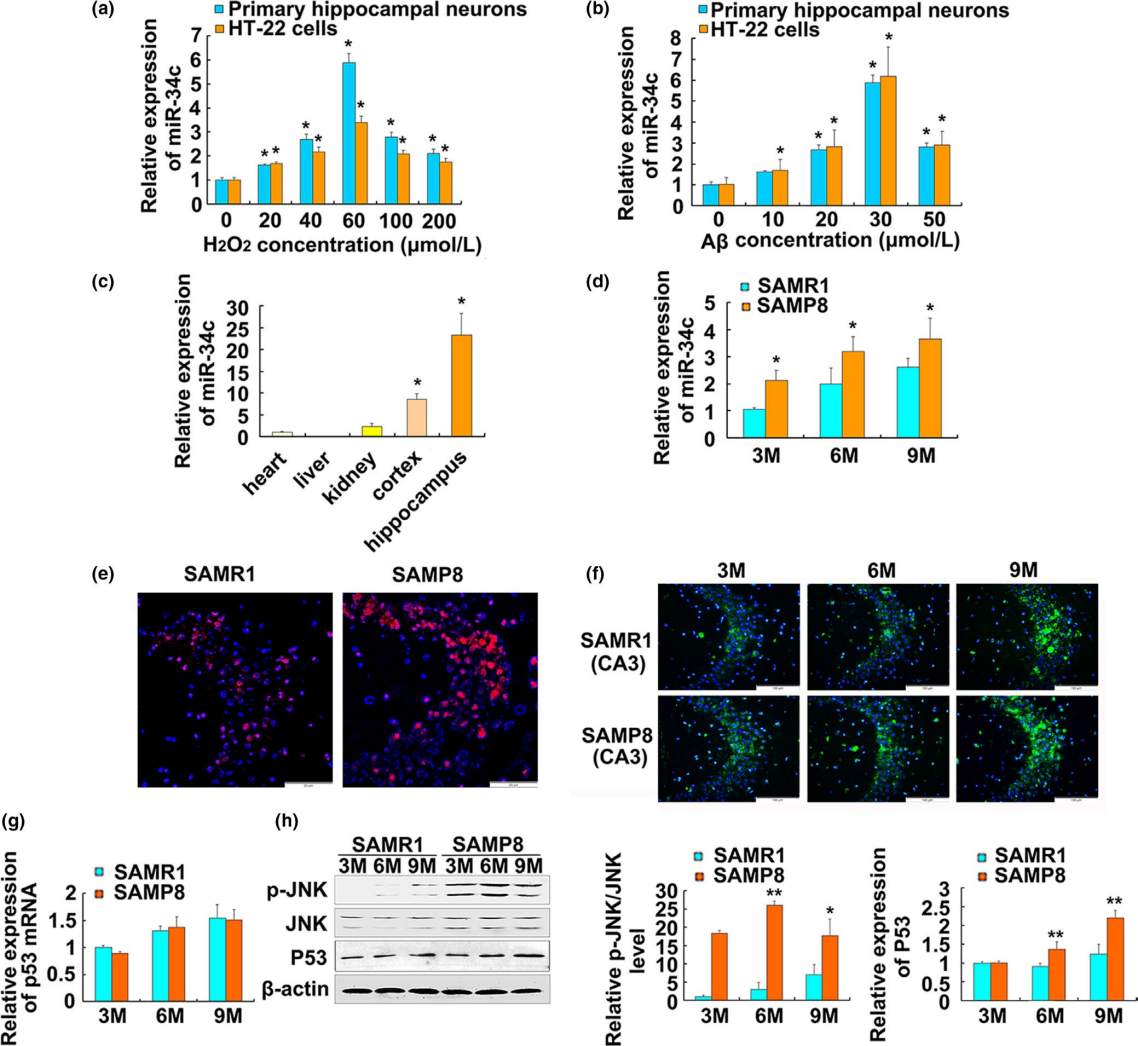
Expression of miR‐34c, ROS generation, JNK activation, and P53 accumulation in hippocampus of SAMP8 mice. (a, b) Quantitative real‐time RT–PCR analysis of miR‐34c expression in mouse primary hippocampal neurons and HT‐22 cells treated with different concentrations of H_2_O_2_ (a) or Aβ (b). *p < .05 versus 0 μmol/l, *n* = 3. (c) Distribution of miR‐34c in different tissues of SAMR1 mice. *p < .05 versus*.* heart, liver, or kidney tissues, *n* = 6. (d) Relative expression levels of miR‐34c in the hippocampus of 3‐, 6‐, and 9‐month‐old SAMP8 and SAMR1 mice. *p < .05 versus age‐matched SAMR1 mice, *n* = 6. All data of miR‐34c expression were normalized to U6‐snRNA expression levels. Error bars represent s.d. of the indicated experiment replicates. (e) DHE fluorescence staining was applied to detect ROS generation in the hippocampus from 6‐month‐old SAMP8 or SAMR1 mice. Scale bars = 25 μm (f) Expression of P53 protein was detected by immunofluorescence in CA3 subfield of the hippocampus from 3‐, 6‐, and 9‐month‐old SAMP8 and SAMR1 mice. Original magnification was 40×. (g) Quantitative real‐time RT–PCR analysis of the p53 mRNA levels in the hippocampus of 3‐, 6‐, and 9‐month‐old SAMP8 and SAMR1 mice, *n* = 6. U6‐snRNA was used as a control for the normalization of samples. (h) Expression changes of p‐JNK/JNK/P53 protein levels in the indicated groups. Western blot analyses are shown in the left panel. Densitometry analysis is shown in the right panel. The β‐actin was used as a loading control. *p < .05 versus age‐matched SAMR1. *n* = 6. Error bars represent s.d. from the mean of indicated independent experiments with similar results

To investigate whether miR‐34c plays functional roles in the pathological process of AD, the expression patterns of miR‐34c were determined in vivo and in vitro. We found the expression levels of miR‐34c were increased in a concentration‐dependent manner both in the oxidative stressed neurons stimulated with H_2_O_2,_ and in the AD cell model stimulated with Aβ42 (Figure 1a,b). The distribution results showed the levels of miR‐34c were significantly higher in cortex and hippocampus than in heart, liver, and kidney tissues in mice (Figure 1c). In addition, the miR‐34c levels were increased gradually in the hippocampus of 3‐, 6‐, and 9‐month‐old SAMP8 mice compared to age‐matched SAMR1 mice (Figure 1d), which validated our previous microarray analysis results. These results suggested that increased miR‐34c might potentially play a key role in the process of neuron injury in AD.

To understand the upstream signaling pathway of increased miR‐34c induced by oxidative stress, we detected the ROS generation, JNK activation, and P53 accumulation in the hippocampus of SAMP8 or SAMR1 mice. The results of DHE staining showed the ROS generation in the hippocampus from 6‐month‐old SAMP8 was increased compared with SAMR1 mice (Figure 1e). The immunostaining results showed that the most P53 proteins were distributed in CA3 subfield of the hippocampus from SAMP8 or SAMR1 mice and were increasing with aging (Figure 1f). Although the differences in relative expression of p53 mRNA were not found between SAMP8 and SAMR1 mice aged 3, 6 and 9 months old (Figure 1g), the JNK activation and P53 accumulation in the hippocampus of SAMP8 were increased compared with SAMR1 mice with aging (Figure 1h). These results suggested that ROS generation, JNK activation, and P53 accumulation are involved in neuron injury in hippocampus of SAMP8 mice.

### 2.2 Increased miR‐34c expression is regulated by ROS‐JNK‐p53 pathway

**Figure 2:**
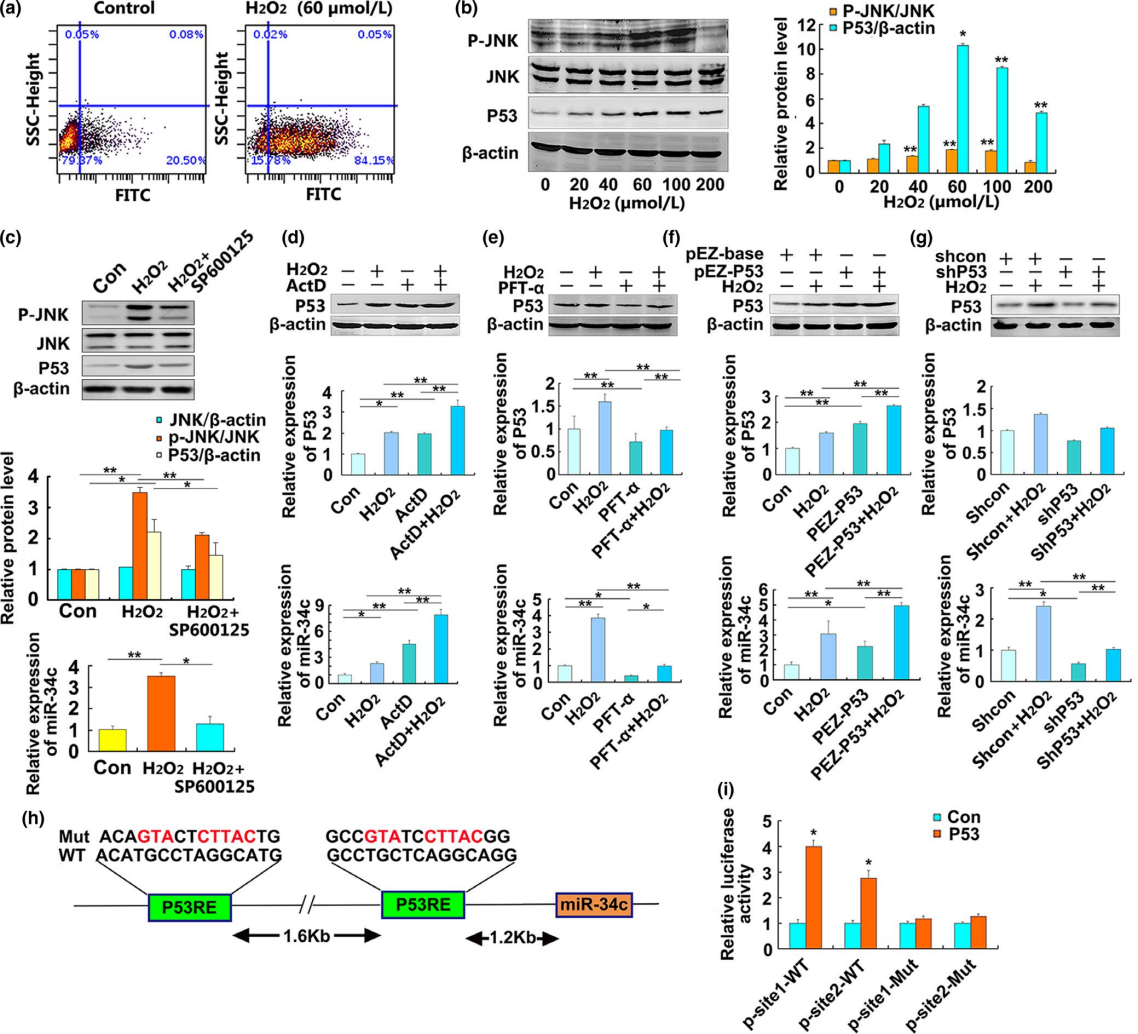
Expression of miR‐34c is regulated by ROS‐JNK‐p53 pathway in neurons. (a) ROS generation was induced by H_2_O_2_ in HT‐22 cells. HT‐22 cells treated with 60 μmol/l H_2_O_2_ for 24 hr, and ROS generation was detected using flow cytometry (FCM). (b) Western blot analysis of p‐JNK, JNK, and P53 protein in the HT‐22 cells exposed to different concentrations of H_2_O_2_ (0, 20, 40, 60, 100, and 200 μmol/l) for 24 hr. Densitometry analysis was performed and normalized to β‐actin. The values are the mean ± *SD* (*n* = 3). *p < .05,**p < .01 versus 0 μmol/l H_2_O_2_ group. (c) Effects of JNK inhibitor SP600125 on p‐JNK, JNK, and P53 protein (top and middle panel) and miR‐34c expression (bottom panel) in HT‐22 cells. HT‐22 cells were treated with SP600125 (50 μmol/l) for 3 hr and then incubated with H_2_O_2_ (60 μmol/l) for 24 hr. The values are the mean ± *SD* (*n* = 3). *p < .05 ** *p* < .01, *n* = 3. (d–g) Changes of P53 protein (top and middle panel) and miR‐34c expression (bottom panel) in the indicated groups. HT‐22 cells were treated with actinomycin D (Act D, 10 nmol/l) (d) or pifithrin‐α (PFT‐α, 10 nmol/l) (e) for 3 hr, or transfected with pEZ‐p53 (f) or shP53 (g) for 24 hr, and then treated with (or without) H_2_O_2_ (60 μmol/l) for 24 hr. All data of P53 levels were normalized to β‐actin, and the relative expression of miR‐34c was normalized to U6‐snRNA expression levels. *p < .05, **p < .01. *n* = 3. (h) The predicted P53 binding sites in the promoter region of miR‐34c and the designed mutant sequences were indicated. (i) Relative luciferase activities of the miR‐34c promoter were measured in the 293A cells cotransfected pEZ‐p53 with wild‐type luciferase vector or mutant luciferase vector. *p < .05 versus Con, *n* = 3

To validate the oxidative stress‐induced increase in miR‐34c is mediated by ROS‐JNK‐P53 pathway, we found that H_2_O_2_ induced an increase in intracellular ROS production in HT‐22 cells by flow cytometry (FCM) assay (Figure 2a) and the JNK activation and P53 accumulation were induced by exogenous H_2_O_2_ in a concentration‐dependent manner (Figure 2b). JNK has been reported to be implicated in P53 accumulation (Chen et al., 2018; Wu et al., 2018). Thus, the effect of JNK on P53 accumulation was determined by using of JNK inhibitor SP600125. We found SP600125 inhibited the phosphorylation of JNK, P53 accumulation, and the increased miR‐34c induced by H_2_O_2_ in HT‐22 cells treated with H_2_O_2_ (60 μmol/l) for 24 hr (Figure 2c). These results suggest that JNK initiates p53 accumulation and activation after H_2_O_2_ treatment in HT‐22 cells. To understand the regulatory effect of P53 protein on the expression of miR‐34c, HT‐22 cells were treated with p53 activator actinomycin D (Act D) or p53 inhibitor pifithrin‐α (PFT‐α) for 3 hr, or transfected with pEZ‐p53 or shP53 for 24 hr, and then, cells were treated with (or without) H_2_O_2_ for 24 hr. We found Act D or p53 over expression mediated P53 protein accumulation and increased the miR‐34c level (Figure 2d,f), but PFT‐α or knockdown of the p53 gene could block the increase in miR‐34c induced by H_2_O_2_ in HT‐22 cells (Figure 2e,g). Bioinformatic analysis suggested that the miR‐34c promoter harbors two potential P53‐binding sites (Figure 2h), and the results of luciferase assays revealed that P53 could increase the luciferase activity of wild‐type miR‐34c promoter reporter vectors but not that of mutant reporter vectors (Figure 2i). It was suggested that the potential P53‐binding sites at Site 1 (−1,216 bp to −1,202 bp) and Site 2 (−2,830 to −2,816 bp) were probably responsible for enhancing miR‐34c transcription.

### 2.3 Expression of SYT1 is downregulated in the hippocampus of SAMP8 mice

**Figure 3:**
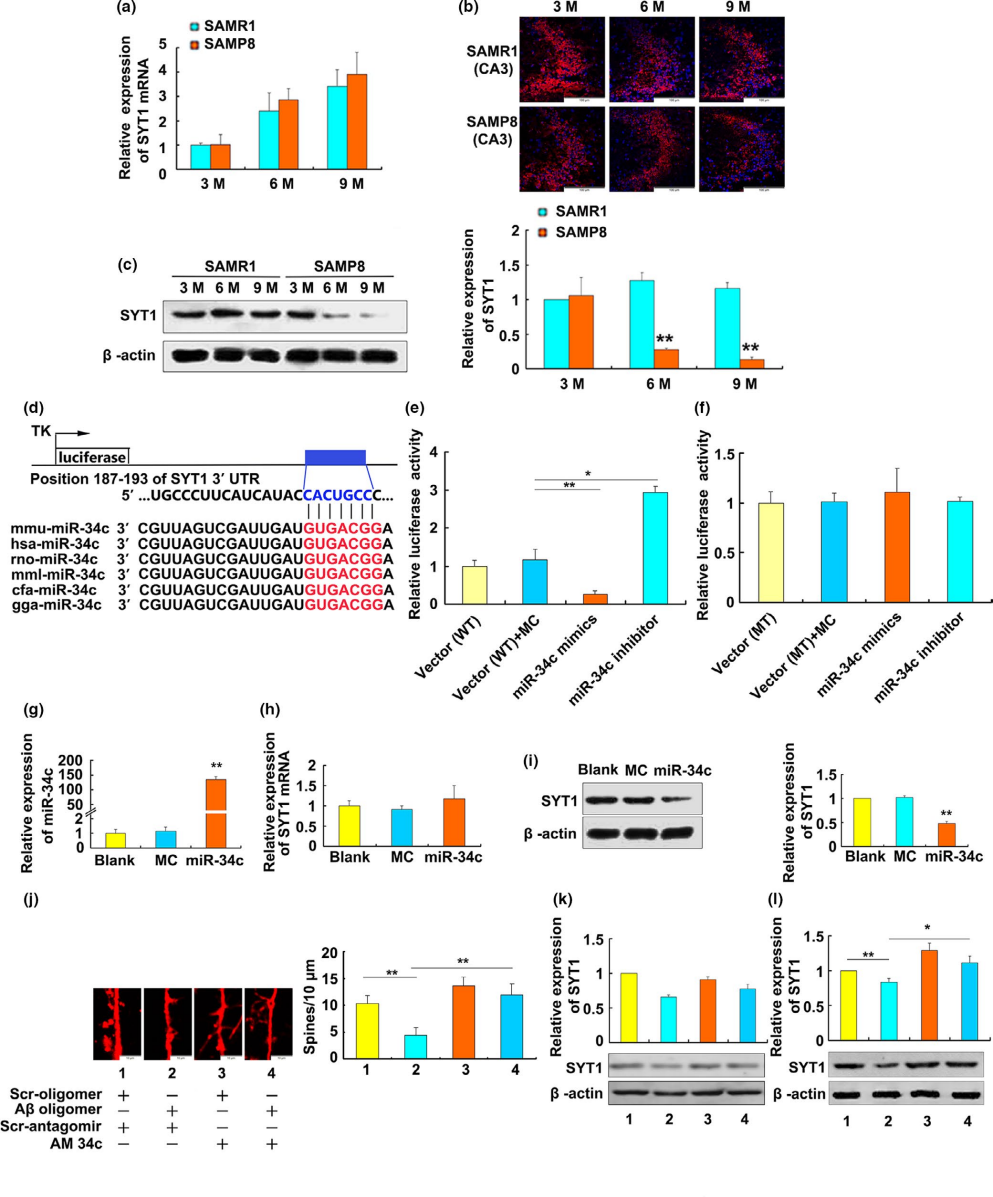
MiR‐34c targets SYT1 transcript through its binding sites. (a) Quantitative real‐time RT–PCR analysis of the SYT1 mRNA levels in the hippocampus of 3‐, 6‐, and 9‐month‐old SAMP8 or SAMR1 mice. U6‐snRNA was used as a control for the normalization of samples (*n* = 6). (b, c) Expression of SYT1 protein in the hippocampus of SAMP8 or SAMR1 mice by immunofluorescence (b) (40×) and Western Blot analysis (c). The β‐actin was used as a loading control. **p < .01 versus*.* age‐matched SAMR1 mice (*n* = 6). 3M, 6M, and 9M stand for 3‐, 6‐, and 9‐month‐old group. (d) The predicted miR‐34c binding site in the 3′‐UTR of SYT1 mRNA and miR‐34c sequences of different species was indicated. (e, f) Luciferase assay of SYT1 3′‐UTR. 293A cells were cotransfected with SYT1 3′‐UTR luciferase vector (WT) and miR‐34c mimic (100 nmol/l) or miR‐34c inhibitor (100 nmol/l) (e), or cotransfected with mutated 3′‐UTR luciferase vector (MT) and miR‐34c mimic (100 nmol/l) or miR‐34c inhibitor (100 nmol/l) (f). Luciferase activities were detected at 24 hr after transfection (*n* = 3). Vector stands for psiCHECK‐SYT1‐UTR transfection only. MC stands for mimics control group. *p < .05, **p < .01. (g–j) Expression of SYT1 is regulated by miR‐34c in HT‐22 cells. MiR‐34c mimics or mimic control (MC) was transfected into HT‐22 cells for 24 hr before harvesting. The levels of miR‐34c (g) and syt1 mRNA (h) were determined by qRT–PCR analysis, and SYT1 protein (i) was detected by Western blot analysis in HT‐22 cells (*n* = 3). **p < .01 versus blank or MC. (j) Effects of miR‐34c inhibitor (AM34c) on dendritic outgrowth in primary hippocampal neurons treated with Aβ oligomer. Primary hippocampal neurons from SAMR1 were treated with AM34c or scrambled antagomir (50 nmol/l). After 48 hr, cells were treated with 5 μmol/l Aβ oligomer or scrambled oligomer. The dendritic spine density was observed after phalloidin staining. Scale bars = 100 μm. (k,l) Effects of miR‐34c inhibitor (AM34c) on the expression of SYT1 in primary hippocampal neurons (k) and HT‐22 cells (l) treated with Aβ oligomer. The relative expression levels of SYT1 protein were detected by Western blot analysis in HT‐22 cells and normalized to β‐actin expression. Densitometry analysis results are shown in the right panel (*n* = 3). **p < .01 versus scrambled oligomer + scrambled antagomir, *p < .05 versus Aβ oligomer + scrambled antagomir

To further speculate the biological functions of miR‐34c in AD, bioinformatics analysis was performed to predict its target genes. Among those computational predicted targets, SYT1 was selected for further validation due to its higher predictive scores and its implications in neurotransmitter release at synapses (Yoshihara & Montana, 2004). We found that the SYT1 mRNA levels were increased gradually with aging, but no significant differences were found between SAMP8 and age‐matched SAMR1 mice (Figure 3a). To address the cell‐type distribution of SYT1 protein in the hippocampus, the immunohistochemical results revealed that the high‐abundance proteins of SYT1 were expressed in CA3 subfields of mouse hippocampus. A gradual decrease in the fluorescence intensity has been observed throughout the hippocampus with aging. However, the abundance of SYT1 proteins in 6‐ and 9‐month‐old SAMP8 mice were lower compared with age‐matched SAMR1 mice (Figure 3b). Western blot analysis results showed that the SYT1 protein levels were significantly downregulated in the hippocampus of 6‐ and 9‐month‐old SAMP8 mice compared with age‐matched SAMR1 mice (Figure 3c). These results indicated that the expression of SYT1 may be downregulated at post‐transcriptional level in hippocampus of SAMP8 mice.

### 2.4 miR‐34c targets SYT1 transcript through its binding sites

Bioinformatic analysis results suggested that the 3'‐UTR of SYT1 mRNA harbors one highly conserved potential binding site of miR‐34c (Figure 3d). To examine the direct binding of miR‐34c to the 3'‐UTR of the SYT1 mRNA, luciferase reporter plasmids were generated containing the miR‐34c‐binding site or mutant sequence. As expected, miR‐34c mimics led to significantly reduced luciferase activity compared to the mimic control, but the inhibitor of miR‐34c increased the luciferase activity obviously (Figure 3e). However, the mutated sequence of the SYT1 3'‐UTR abrogated the repressive effects of miR‐34c on the luciferase activity of its target 3'‐UTR (Figure 3f). These results indicated that miR‐34c directly binds to the 3'‐UTR of SYT1 mRNA sequences.

To further validate whether miR‐34c could regulate the translation of SYT1 protein, miR‐34c mimics or a mimic control was transfected to HT‐22 cells. The results of real‐time PCR showed that miR‐34c mimics leads to a significant increase in miR‐34c levels compared with the mimic control (Figure 3g). However, the SYT1 mRNA levels in HT‐22 cells were not affected by miR‐34c mimic transfection (Figure 3h). Meanwhile, miR‐34c mimic overexpression led to a significant decrease in the SYT1 protein levels compared with the mimic control (Figure 3i). Syt1 overexpression could in turn decrease the expression of miR‐34c, and syt1 knockdown could increase the expression of miR‐34c (Figure S1). To test the effects of miR‐34c inhibitor (AM 34c) on the decrease in dendritic spine density induced by Aβ, rhodamine‐conjugated phalloidin staining results suggested treatment of primary mouse hippocampal neurons with Aβ oligomer caused a decrease in the density of dendritic spines, but AM 34c rescued the Aβ‐induced damage in the dendritic spines (Figure 3j). Western blot results also revealed AM34c increased the expression levels of SYT1 protein compared with Aβ oligomer treatment in primary hippocampal neurons and HT‐22 cells (Figure 3k and 3l). These results suggested that miR‐34c plays a functional role in regulation of SYT1 expression in neurons.

### 2.5 miR‐34c regulates the expression of SYT1 in SAMP8 mice

**Figure 4:**
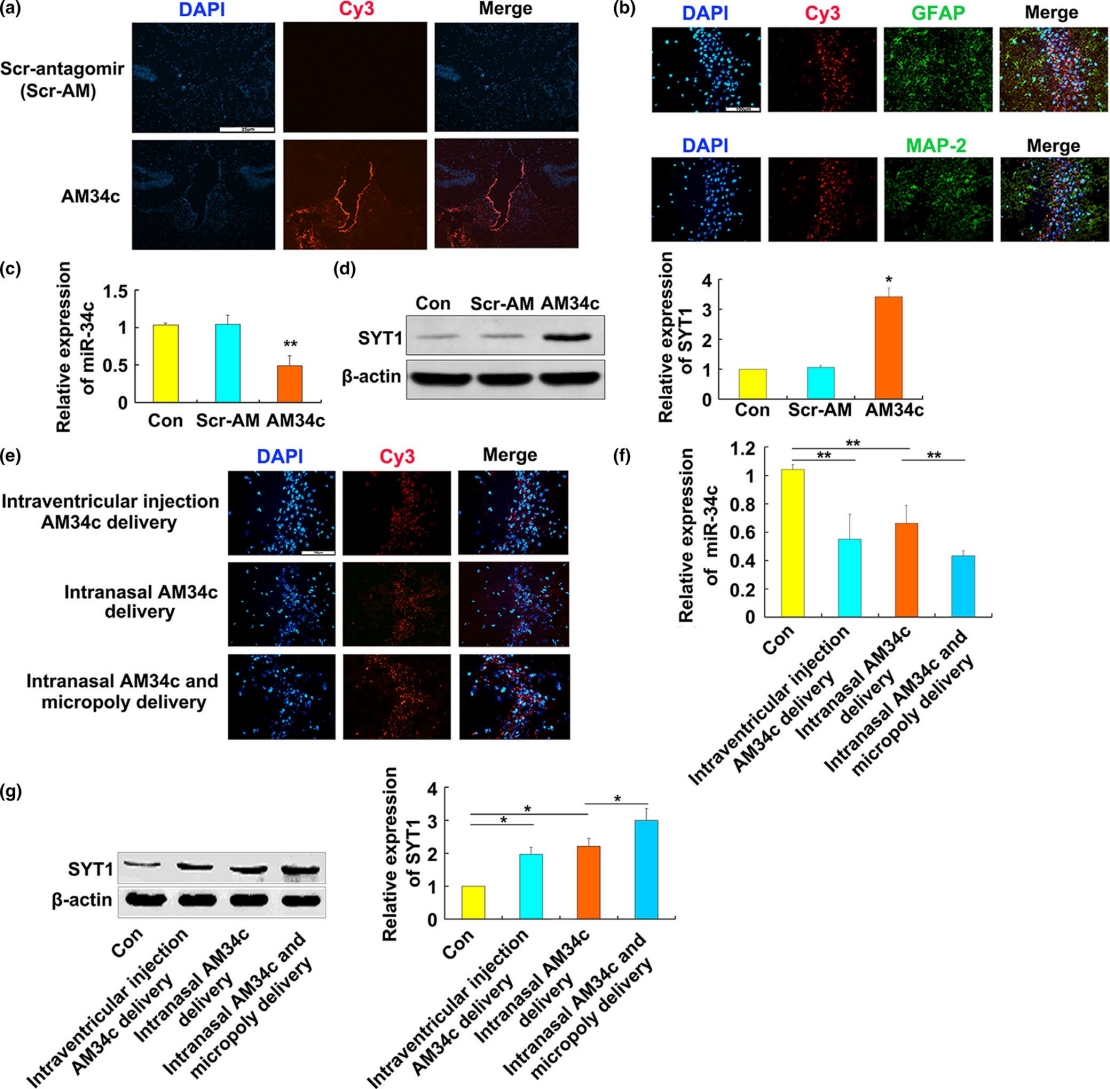
SYT1 protein levels were downregulated by delivery of miR‐34c inhibitor in SAMP8 mice. (a) Intraventricular injection of Cy3‐labeled AM34c (Cy3‐AM34c). Six‐month‐old SAMP8 were injected with Cy3‐AM34c in the third ventricle (*n* = 6). At 24 hr, fluorescence for Cy3‐AM34c was distributed throughout the hippocampus and surrounding tissues. Scale bars = 25 μm. (b) Cy3‐AM34c was uptaken by MAP2‐positive neurons and GFAP‐positive glial cells in the CA3 subfield of hippocampus. Scale bars = 100 μm. (c) The relative miR‐34c levels in the hippocampus of SAMP8 mice after antagomir delivery. U6‐snRNA was used as a control for the normalization of samples (*n* = 6). **p < .01 versus Scr‐AM. (d) AM34c significantly increased the level of SYT1 protein in the hippocampus of SAMP8 mice. Western blot analysis was performed to detect the hippocampal SYT1 protein in SAMP8 mice injected with AM34c or the scramble antagomir (Scr‐AM) at 3 weeks after the injection (*n* = 6). *p < .05 versus Scr‐AM. (e) Distribution of Cy3‐labeled AM34c in the hippocampus of SAMP8 mice through different delivery ways. Cy3‐AM34c was delivered by intraventricular injection or intranasal delivery with or without Micropoly‐transfecter^TM^ Tissue Reagent in SAMP8 mice. At 24 hr after delivery, the distribution of Cy3‐AM34c was observed in the CA3 subfields of hippocampus by fluorescence microscope (*n* = 6). Scale bars = 100 μm. (f) The relative expression of miR‐34c in the hippocampus of SAMP8 mice after AM34c delivery through different ways. U6‐snRNA was used as a control for the normalization of samples (*n* = 6). **p < 0.01*vs* indicated group. (g) STY1 protein levels in hippocampus of SAMP8 mice after miR‐34c antagomir delivery through three different ways. β‐actin was used as a control for the normalization of samples. *p < .05 versus indicated group. The values are the means ± S.D. (*n* = 6)

To investigate the functional roles of miR‐34c in AD, a Cy3‐labeled miR‐34c inhibitor AM34c (Cy3‐AM34c) was injected into the third ventricle of 6‐month‐old SAMP8 mice. We found that the Cy3‐AM34c was distributed throughout the hippocampus and surrounding tissues after 24 hr (Figure 4a). Fluorescence‐staining results showed that Cy3‐AM34c was taken up by MAP2‐positive neuronal cells and glial fibrillary acidic protein (GFAP)‐positive glial cells in the CA3 subfield of hippocampus (Figure 4b). The miR‐34c levels were decreased, but the SYT1 protein levels were increased significantly in the hippocampus after AM34c delivery at 3 weeks after the injection (Figure 4c,d), which suggested that SYT1 expression was downregulated by miR‐34c in vivo*.* As the intraventricular injection is an invasive method of drug delivery, we investigated whether the antagomir can be delivered via intranasal administration route as well. Indeed, intranasally delivered Cy3‐AM34c was detected in the CA3 subfields of hippocampus of SAMP8 mice after 24 hr and achieved similar effect as intraventricular injection (Figure 4e). Further, we found intranasal delivery of Cy3‐AM34c together with Micropoly‐transfecter significantly facilitated the Cy3‐AM34c diffusion and increased the transfection efficiency compared to intranasal Cy3‐AM34c delivery alone (Figure 4e). Finally, the three ways of the AM34c delivery led to a significant decrease in miR‐34c levels (Figure 4f) as well as a significant increase in SYT1 protein levels compared with the control mice (Figure 4g). These results suggested that intranasal delivery of the antagomir to the brain is feasible.

### 2.6 Administration of AM34c enhances memory function in SAMP8 mice

**Figure 5:**
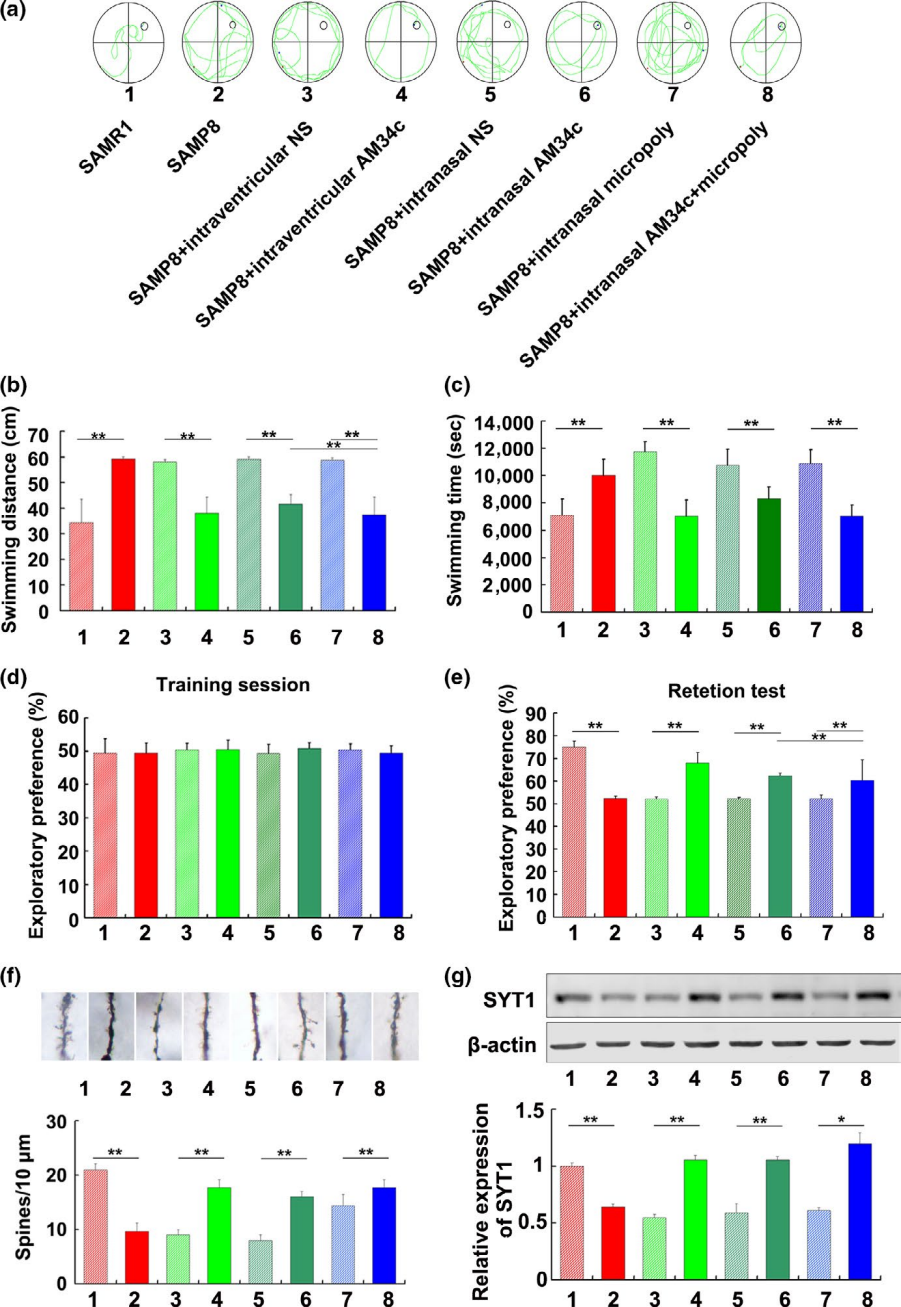
Administration of AM34c enhanced memory function and synaptic function in SAMP8 mice. (a) The representative swimming path for SAMP8 mice to find the platform in Morris water maze test after administration of AM34c through different ways. (b) Swimming distance and (c) swimming time results from Morris water maze test of 6‐month‐old SAMP8 mice after delivery of AM34c by different ways for 3 weeks. **p < .01 versus indicated group (*n* = 6). (d, e) Novel object recognition (NOR) results from Morris water maze test of 6‐month‐old SAMP8 mice after delivery of AM34c by different ways for 3 weeks. Data shown are percent novel object recognition in the training session (d) and test session (e). The values are the means ± S.D. **p < .01 versus indicated group (*n* = 6). (f) Golgi staining in hippocampus of 6‐month‐old SAMP8 mice after delivery of AM34c by different ways for 3 weeks. Scale bar = 5 µm. **p < .01, versus indicated group (*n* = 6). (g) STY1 protein levels in hippocampus of 6‐month‐old SAMP8 mice after delivery of AM34c by different ways for 3 weeks. *p < .05, **p < .01 versus indicated group

To investigate whether AM34c administration improves the cognitive deficits in SAMP8 mice, memory function of 6‐month‐old mice was examined by the MWM test and NOR test after administration of AM34c by different delivery ways for 3 weeks (Figure 5a). The results of MWM test showed that intraventricular injection or intranasal delivery of AM34c markedly reduced the averaged swimming distance (Figure 5b) and swimming time (Figure 5c) in SAMP8 mice compared with the control group. It is worth mentioned that intranasally administered AM34c together with Micropoly‐transfecter achieved better effects compared with intranasal AM34c delivery alone in SAMP8 mice (Figure 5b,c). Results of NOR test also identified that administration of AM34c by the three delivery ways significantly improved cognitive deficits in SAMP8 mice (Figure 5d,e). The results of Golgi staining revealed that intraventricular injection or intranasal delivery of AM34c rescued dendritic spine deterioration, but AM34c together with Micropoly‐transfecter achieved similar effects compared with intranasal AM34c delivery alone (Figure 5f). In addition, AM34c delivery increased the protein levels of SYT1; moreover, AM34c together with Micropoly‐transfecter achieved better effects compared with intranasal AM34c delivery alone (Figure 5g). These results suggested that inhibition of miR‐34c improved memory decline in SAMP8 mice.

### 2.7 The levels of circulating miR‐34c are increased in patients with aMCI

**Figure 6:**
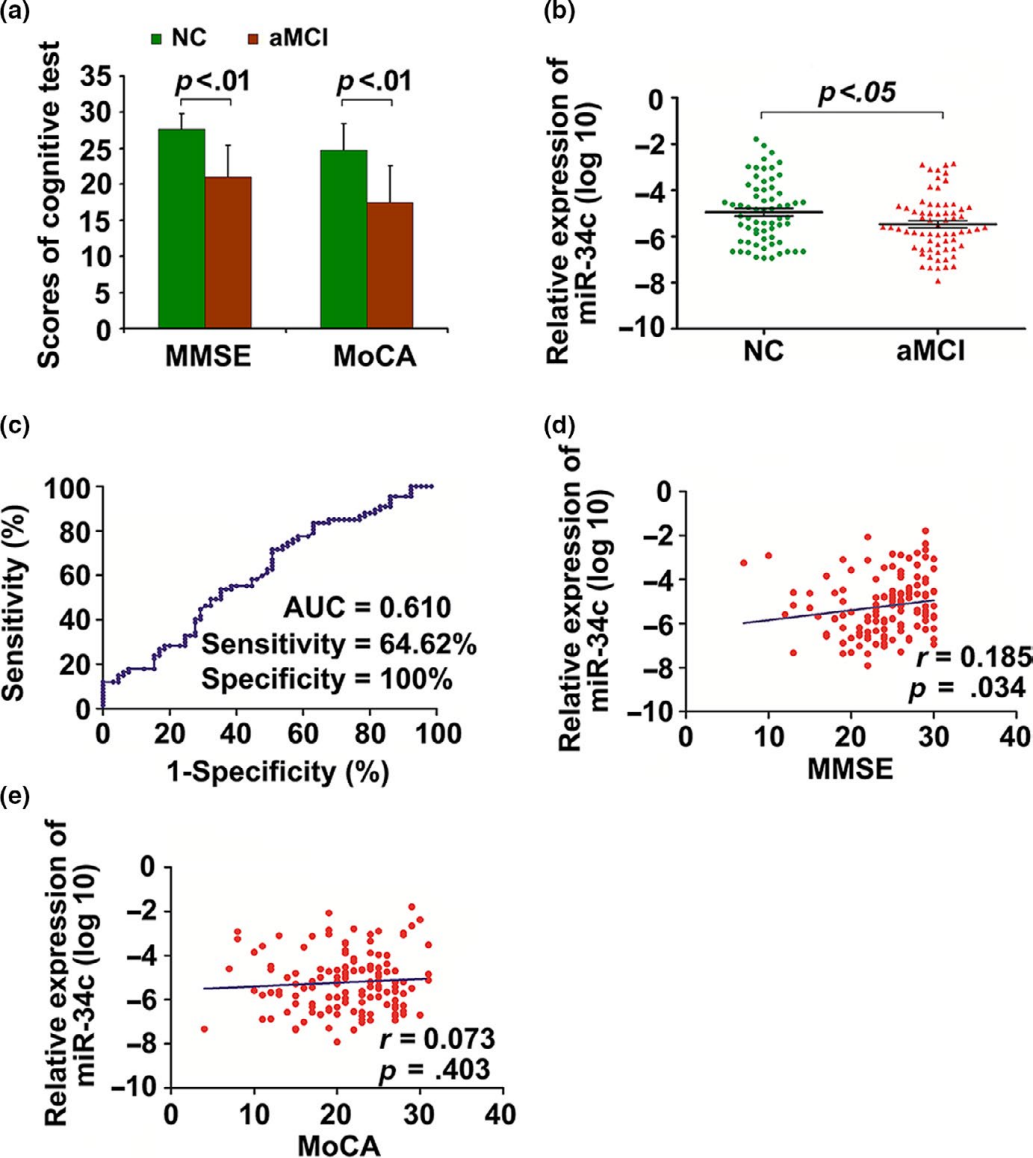
The expression levels of serum miR‐34c are increased in patients with aMCI. (a) MMSE and MoCA scores of the surveyed samples. (b) The relative expression levels of serum miR‐34c in patients with aMCI and normal controls. The 2^−ΔΔ^ *^C^* ^t^ method was used to determine the relative expression of serum miR‐34c levels for the surveyed samples, and the Δ*C*_t_ value of mi‐34c was transformed into log10^−2 ‐ΔCt^ as ordinate for statistical analyzed. (c) ROC curve of serum miR‐34c is established to distinguish aMCI from normal controls. (d, e) The correlations between serum miR‐34c levels and MMSE (c) or MoCA (d) scores by Spearman's correlation coefficient

To determine the serum miR‐34c levels in patients with aMCI, we included 71 patients with aMCI and 69 normal age‐matched controls. Among aMCI patients, 32 were males and 39 were females, and the mean age was 73.89 ± 6.43 years. Among the controls, 27 were males and 42 were females, and the mean age was 74.17 ± 6.87 years. There were no significant statistical differences of age and gender between aMCI group and control group (Table S1). As shown in Figure 6a, MMSE and MoCA scores of the aMCI patients were lower than that of normal age‐matched controls. The serum levels of miR‐34c were significantly increased in patients with aMCI compared with normal age‐matched controls (Figure 6b). To evaluate the predictive value of circulating miR‐34c levels for diagnosis of aMCI, ROC analysis was performed and the results showed that the serum miR‐34c had an AUC of 0.610 (95% CI: 0.514–0.706) (Figure 6c). The optimal cutoff point for serum miR‐34c was −5.02, with 64.62% sensitivity and 100.0% specificity. Next, we analyzed the potential correlations between serum miR‐34c levels and MMSE/MoCA scores. The results of Spearman's correlation analysis showed a positive correlation between the relative expression of serum miR‐34c and MMSE scores (*r* = .185, *p* = .034) (Figure 6d), but the levels of serum miR‐34c had no significant correlation with MoCA scores (*r* = .073, *p* = .403) (Figure 6e).

## 3 DISCUSSION

Alzheimer's disease (AD) is an aging‐related neurodegenerative disease and accounts for majority of human dementia. Cancer is another disorder prevalent in the elderly, and it is characterized by uncontrolled cell proliferation. Some epidemiological researches have reported the negative association between AD and cancer. Understanding the relationship between AD and cancer, in terms of cellular pathways and molecular mechanisms, will help to open up important new areas of prevention and treatment research for both diseases. It is notable that many factors are downregulated in cancer to inhibit growth and survival, but are upregulated in AD contributing to neurodegeneration. A broad spectrum of miRNAs has been identified to be involved in the etiology of AD and cancer. Among them, miR‐34c, as a tumor suppressor and a critical mediator in the p53 pathway (Beard et al., 2016; Cortez et al., 2016), can reduce cell growth, induce apoptosis, and affect cell migration (Tao et al., 2016; Yang et al., 2015). It is reported that the levels of miR‐34c were increased in circulating blood samples of patients with AD compared to age‐matched normal controls (Bhatnagar et al., 2014)^.^ Our previous research also indicated that miR‐34c was upregulated significantly in the SAMP8 mouse model by miRNA microarray analysis (Zhou et al., 2016). In current study, we confirmed miR‐34c was increased in a concentration‐dependent manner by stimulation with H_2_O_2_ or Aβ in hippocampal neurons. We also confirmed that miR‐34c was upregulated in the hippocampus of SAMP8 mice with aging. Furthermore, miR‐34c was highly expressed in the hippocampus and cortex compared to other tissues. These results suggested that increased miR‐34c was involved in oxidative stress process and might potentially play key roles in the pathological process of AD, but its upstream regulatory signaling pathways and the downstream molecular mechanisms in AD remain unclear.

The miR‐34 family (miR‐34a, miR‐34b, and miR‐34c), which is described as a p53 effector, has anti‐proliferative and pro‐apoptotic functions(Beard et al., 2016; Cortez et al., 2016) It is well known that p53 is upregulated in AD brain and leads to neuronal loss, but we do not know whether miR‐34c could be transcriptionally activated by p53 during the progression of AD. In this study, we firstly found that ROS generation, JNK activation, and P53 accumulation were elevated in hippocampus of SAMP8 mice, suggesting ROS‐JNK‐P53 pathway is involved in the pathological process of AD. Then, we revealed that H_2_O_2_ induced an increase in intracellular ROS production and initiated ROS‐JNK‐P53 pathway in HT‐22 cells. Finally, we confirmed P53 protein directly bound to its binding sites (regulatory elements) in the region of miR‐34c promoter and activated the expression of miR‐34c. These results indicated that increased levels of miR‐34c expression were regulated by ROS‐JNK‐p53 pathway in development of AD.

Although miR‐34c, as a tumor suppressor, plays critical roles in cancer, such as reduce cell growth, induce apoptosis, and affect cell migration, however, the pathological roles of increased miR‐34c in AD remain unclear. TargetScan analysis indicates that SYT1 may be a potential target of miR‐34c. SYT1 is a member of a synaptotagmin (syt) family, who plays a critical role in the release of neurotransmitters from presynaptic nerve terminals (Yoshihara & Montana, 2004). It has been reported that SYT1 was required for rapid neurotransmitter release in mouse hippocampal neurons (Evans, Ruhl, & Chapman, 2015)and was decreased in several brain regions and increased in cerebrospinal fluid of patients with Alzheimer's disease (Ohrfelt et al., 2016; Yoo, Cairns, Fountoulakis, & Lubec, 2001). Consequently, SYT1 was selected from the predicted targets of miR‐34c for further validation in this study. Consistent with previous results, SYT1 was highly expressed in the CA3 subfield of the hippocampus, an area that is closely related to learning and memory, and the SYT1 protein levels were decreased in the hippocampus of 6‐ and 9‐month‐old SAMP8 mice, which showed a contrary expression pattern with miR‐34c mRNA. We speculated that miR‐34c might inhibit the translation of SYT1 at the post‐transcriptional level. We further confirmed a direct interaction between miR‐34c with the 3′‐UTR of SYT1 mRNA, which provided a direct evidence for SYT1 being targeted by miR‐34c. Syt1 overexpression could in turn decrease the expression of miR‐34c, and syt1 knockdown could increase the expression of miR‐34c, but the underlying mechanism still needs further study. Moreover, we also confirmed that miR‐34c downregulated SYT1 expression at the post‐transcriptional level in vitro and the inhibitor of miR‐34c rescued the Aβ‐induced decrease in the density of dendritic spines in primary hippocampal neurons. These results suggested that miR‐34c may play critical roles in the synaptic injury through post‐transcriptional regulation of SYT1 expression during AD development.

MiRNAs have been identified as potential biomarkers and therapeutic targets in cancer and other disease (Cheng et al., 2015; Schmidt et al., 2018; Zeng et al., 2017). Then, whether administration of miR‐34c inhibitor could improve the cognitive deficits in AD model? We firstly confirmed that the AM34c delivery by different ways achieved similar effects on Cy3‐AM34c distribution surrounding the hippocampus tissues and led to a significant decrease in miR‐34c levels as well as a significant increase in SYT1 protein levels compared with the control mice. The results of MWM test and NOR test also identified that administration of miR‐34c inhibitor by three delivery ways significantly improved cognitive deficits in SAMP8 mice. These results suggested that downregulation of miR‐34c improved memory decline in SAMP8 mice, and this effect depended at least partly on the post‐transcriptional regulation of SYT1 expression by miR‐34c.

It is reported that the levels of miR‐34c were significantly increased in circulating blood samples of patients with AD and it might be a potential biomarker for sporadic AD. Patients with AD gradually lose their cognitive competence in the course of the disease, and aMCI is a transitional stage between normal aging and dementia, representing AD’s early stage. To the best of our knowledge, no reports were found about serum miR‐34c levels in patients with aMCI. In the current study, we found the serum levels of miR‐34c were significantly increased in patients with aMCI compared with normal age‐matched controls and the ROC analysis results indicated that the circulating miR‐34c levels might be a predictive biomarker for diagnosis of aMCI with 64.62% sensitivity and 100.0% specificity. Further, the results of Spearman's correlation analysis showed a positive correlation between the relative expression of serum miR‐34c and MMSE scores, but the levels of serum miR‐34c had no correlation to MoCA scores. Future studies with larger age‐matched cohorts will validate these results and reveal whether the miR‐34c change is characteristic of sporadic AD as a biomarker criterion.

In conclusion, the tumor suppressor miR‐34c was upregulated through ROS‐JNK‐p53 pathway in development of AD. It plays critical roles in decrease of synaptic function by post‐transcriptional regulation of SYT1, and inhibition of miR‐34c improved memory decline in AD model. The serum levels of miR‐34c were significantly increased in patients with aMCI, and it might be a predictive biomarker for diagnosis of aMCI in clinic. The results of this study provided evidence to elucidate the underlying mechanisms of AD pathogenesis, and further understanding the relationship between AD and cancer will open up important new areas of prevention and treatment research for both diseases.

## 4 EXPERIMENTAL PROCEDURES

The detailed procedures and information are described in the Supplement.

### 4.1 Cell culture

Primary hippocampal neurons were cultured as previously described (Zhang et al., 2014). HT‐22 cells were purchased from Life Technologies, and human embryonic kidney 293A cells were purchased from American Type Culture Collection (ATCC).

### 4.2 Animals

SAMP8 and its control strain antisenescence‐accelerated mouse (SAMR1) mice aged 3‐, 6‐, and 9‐ months were obtained from the Animal Center of Beijing University Medical Department. All experimental procedures were approved by the Animal Care and Use Committee of the First Hospital of Hebei Medical University.

### 4.3 Dual luciferase assay

To confirm P53 could bind to the region of miR‐34c promoter, 293 cells were cotransfected with wild‐type or mutant miR‐34c‐promoter‐site1‐Luc or miR‐34c‐promoter‐site2‐Luc combined with pEZ‐p53, or a control vector (GeneCopoeia). To confirm the miR‐34c could bind to the 3’‐UTR of SYT1 mRNA, 293 cells were cotransfected with wild‐type or mutant SYT1 3’‐UTR plasmid combined with miR‐34c mimic or miR‐34c inhibitor. Cells were harvested, and the cell lysates were used for measurement of firefly and Renilla luciferase activities using a dual luciferase reporter assay kit (Promega, Madison, WI, USA) according to the manufacturer's protocol. The normalized values (Renilla/firefly or firefly/Renilla activity) were used for analysis.

### 4.4 Treatment of SAMP8 mice with miR‐34c antagomirs

For brain stereotaxic injection, mice were anesthetized and holes were drilled above the third ventricle (bromega 2.3 mm, L:1.8 mm, V:2.0 mm). Control antagomirs or miR‐34c antagomir (0.5 nmol) was injected into third ventricle over 5 min by the use of a dental drill (Cheng et al., 2007). For intranasal administration of AM34c, anesthetized mice were placed in a supine position with the head in an upright position. AM34c (or Cy3‐labeled AM34c; 5 nmol in 24 µl of 0.1% vol/vol diethylpyrocarbonate‐treated distilled water) was administered together with or without Micropoly‐transfecter™ Tissue Reagent (Micropoly) by pipette in 4 µl drops (total 6 fractions), alternating between each nostril every 2 min. Control mice received an equal volume of vehicle.

### 4.5 Morris water maze (MWM) testing

The Morris water maze test was carried out 1 month after treatment of AM34c by different delivery ways. Briefly, the mice underwent four successive trials a day for 5 days to find a platform hidden 1 cm under water. A digital tracking device was connected to a computer and was used to track the movement of mice in the pool. On the sixth day, the hidden platform was removed. The swimming distance and swimming time were measured.

### 4.6 Novel object recognition test

Memory, attention, and discriminative abilities were tested with the novel object recognition (NOR) test (Cramer et al., 2012). Mice were tested on the second day following the end of the reversal phase of MWM training, as described in Supplement.

### 4.7 Subjects and serum samples

The study of diagnosis and treatment of senile dementia in Hebei Province; URL: http://www.chictr.org.cn/showproj.aspx?proj=8194; Registration number: ChiCTR‐RRC‐11001345. We recruited 71 patients with aMCI and 69 normal controls from our previous cross‐sectional cohort study of aMCI in Hebei province (Xie, Liu, Jiang, Liu, et al., 2017a; Xie, Liu, Liu, Jiang, et al., 2017b; Xie, Xu, Liu, Liu, et al., 2017c). All subjects were aged 60 years and older, and aMCI and controls were matched for age and gender.

### 4.8 Ethics statement

This study was conducted according to the principles of the Declaration of Helsinki (http://www.wma.net/en/30publications/10policies/b3/index.html) and was authorized by the Ethics Committee of the First Hospital of Hebei Medical University. All procedures were carried out with the adequate understanding and written consent of the subjects. After obtaining the inform consents, venous blood (5 ml) was collected from aMCI patients and normal controls at the First Hospital of Hebei Medical University.

### 4.9 Statistical analysis

Statistical analyses were performed by SPSS software version 16.0 (SPSS, Inc.). Relative expression levels of miRNAs were calculated by the 2^−Δ^ *^C^* ^t^ method. Quantitative data were compared with *t* test between two groups. For categorical variables, the chi‐square test was used. Spearman's correlation coefficient was calculated to estimate the correlations between miRNAs levels and MMSE or MoCA scores. Receiver operating characteristic (ROC) curves were constructed, and the areas under curves (AUC) were analyzed to evaluate the diagnostic performance of each miRNA. All test were two‐sided, and *p* < .05 was considered as statistically significant.

## CONFLICTS OF INTEREST

The authors declare that they have no conflicts of interest.

## AUTHOR CONTRIBUTIONS

S.X. and R.Z. designed and supervised the study, K.Z., H.Z., and Y.Y. performed the molecular biological experiments and morphological experiment; L.J. helped on animal maintenance and animal experiments, and behavioral tests; R.W. and W.X. performed the AD patient experiments, B.X. and Z.G. performed the statistical analysis; D.C. provided technical support; and S.X., Z.S., and R.Z. wrote the manuscript.

## Data Availability

The data that support the findings of this study are available on request from the corresponding author. The data are not publicly available due to privacy or ethical restrictions.
