# Erratum to: Increased miR‐34c mediates synaptic deficits by targeting synaptotagmin 1 through ROS‐JNK‐p53 pathway in Alzheimer's disease

**DOI:** 10.1111/acel.13933

**Published:** 2023-08-01

Shi, Z, Zhang, K, Zhou, H, et al. Increased miR‐34c mediates synaptic deficits by targeting synaptotagmin 1 through ROS‐JNK‐p53 pathway in Alzheimer's Disease. *Aging Cell*. 2020; 19:e13125. https://doi.org/10.1111/acel.13125

In the published version of Shi, et al. (2020), the titles of the vertical coordinates of Figure 5(b) and (c) were interchanged, the revised version of Figure 5 and its caption is shown below:

**FIGURE 5:**
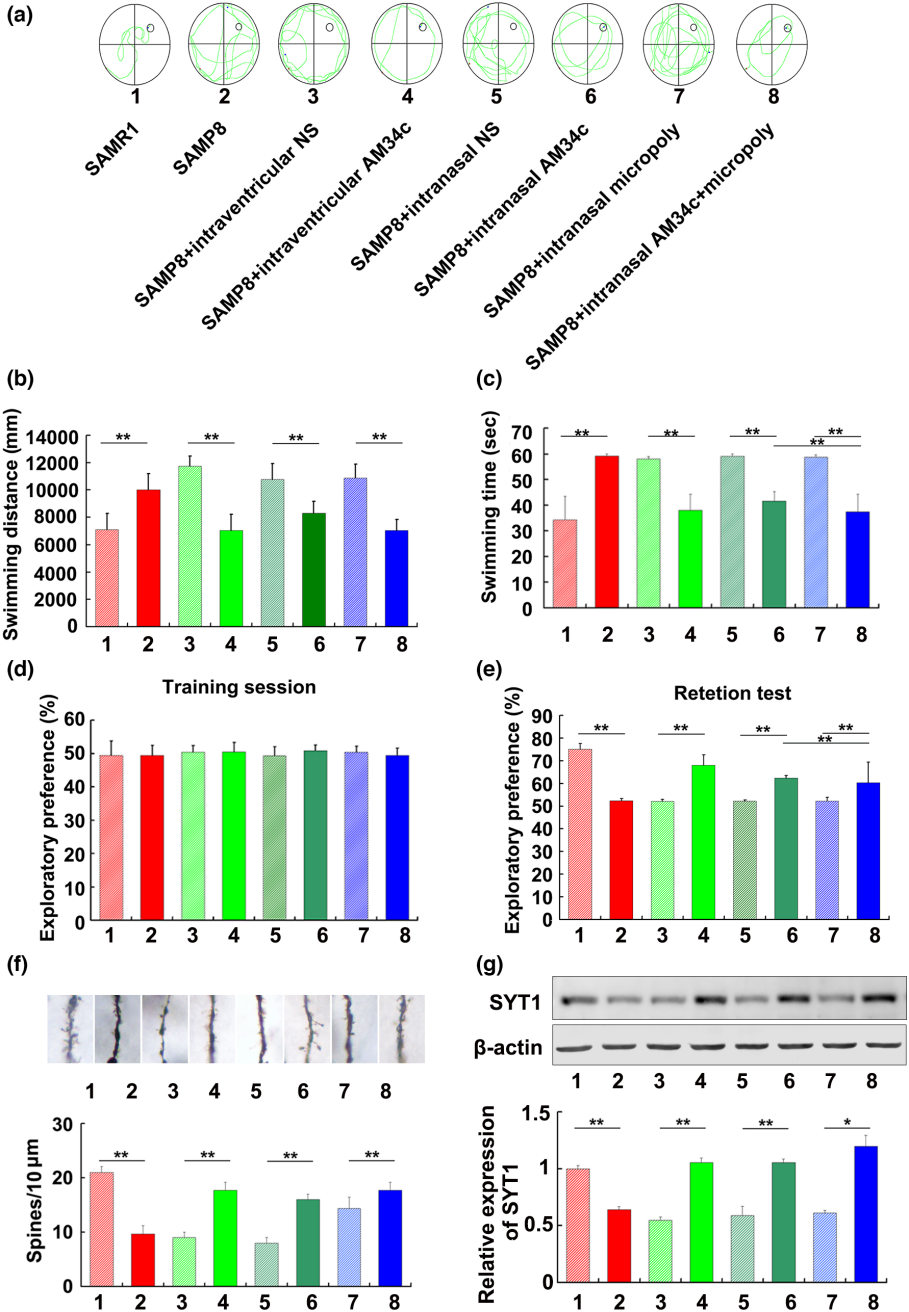
Administration of AM34c enhanced memory function and synaptic function in SAMP8 mice. (a) The representative swimming path for SAMP8 mice to find the platform in Morris water maze test after administration of AM34c through different ways. (b) Swimming distance and (c) swimming time results from Morris water maze test of 6‐monthold SAMP8 mice after delivery of AM34c by different ways for 3 weeks. **p < 0.01 versus indicated group (*n* = 6). (d, e) Novel object recognition (NOR) results from Morris water maze test of 6‐month‐old SAMP8 mice after delivery of AM34c by different ways for 3 weeks. Data shown are percent novel object recognition in the training session (d) and test session (e). The values are the means ± SD **p < 0.01 versus indicated group (*n* = 6). (f) Golgi staining in hippocampus of 6‐month‐old SAMP8 mice after delivery of AM34c by different ways for 3 weeks. Scale bar = 5 μm. **p < 0.01, versus indicated group (*n* = 6). (g) STY1 protein levels in hippocampus of 6‐month‐old SAMP8 mice after delivery of AM34c by different ways for 3 weeks. *p < 0.05, **p < 0.01 versus indicated group.

