# Supplementary material for: Increased miR‐34c mediates synaptic deficits by targeting synaptotagmin 1 through ROS‐JNK‐p53 pathway in Alzheimer’s Disease
Source: Aging Cell. 2020 Feb 24;19(3):e13125. doi: 10.1111/acel.13125 (PMC7059146; doi:10.1111/acel.13125)
Supplement: Supplementary file 1 [file ACEL-19-e13125-s001.doc]

**Supplementary Methods and Materials**

**Cell treatments**

Cells were cultured in complete Dulbecco’s Modification of Eagle’s Medium (DMEM) supplemented with 10% fetal bovine serum. Hydrogen peroxide (H_2_O_2_) was obtained from Sigma-Aldrich (St. Louis, MO, USA). Aβ_42_ peptide was purchased from Millipore (Millipore, Billerica, MA). JNK inhibitor SP600125 (50 μM) was purchased from MedChemexpress (MCE, China). To induce p53 expression, cells were pretreated with actinomycin D (ActD, 10 nM; Cayman Chemical, Michigan, USA), used as a p53 activator. Meanwhile, p53 inhibitor Pifithrin-α (PFT-α, 10 nM; Tocris Bioscience, MN, USA) was used to down-regulate the expression of p53 protein.

**Determination of ROS generation**

Dihydroethidium (DHE, ThermoFisher，Massachusetts, USA) staining was applied to detect ROS generation in the hippocampus from 6-month-old SAMP8 and SAMR1 mice. The intracellular ROS production in HT-22 cells treated with H_2_O_2_ was measured with 2′,7′-dichlorodihydrofluorescein diacetate (DCFH-DA) (solarbio, Beijing, China) probe. DCFH-DA was discarded and the cells were analyzed using BD FACS Calibur Flow Cytometer (BD, New Jersey, USA) with Novo Express software.

Quantitative real-time RT-PCR assay

Total RNA was isolated from the hippocampus or other tissues of SAMR1 and SAMP8 or cultured HT-22 cells with Easteptm Super Total RNA Extraction kit (Promega, Madison, WI, USA), and serum miRNAs were isolated using miRNeasy Serum/Plasma Kit (Qiagen, Germany), according to the manufacturer’s instructions. cDNA synthesis was performed with 300 ng of total RNA or small RNA, using Goscript Reverse Transcriptase Mix (Promega, Madison, WI, USA) and All-in-one^TM^ miRNA First-strend cDNA synthesis kit (GeneCopoeia, Guangzhou, China), respectively. For serum miRNA assay, reverse transcription was carried out using the TaqMan microRNA Reverse Transcription Kit (Applied Biosystems, Foster City, CA, USA). Quantitative real-time RT-PCR (qRT-PCR) was used to measure the genes. The qRT-PCR was performed on 7500 Real Time PCR System (Applied Biosystems, MA, USA) to detect mRNA levels using Gotaq@qpcr MASTER Mix (Promega, Madison, WI, USA) and miRNA levels using All-in-oneT^M^ miRNA qRT-PCR detection kit (Guangzhou, China) with GAPDH or U6 as endogenous control genes, respectively. The detection of serum miR-34c levels were carried out using TaqMan Universal Master Mix II (Applied Biosystems, Foster City, CA, USA). The 2^−ΔΔCt^ method was used to determine the relative expression abundance for the surveyed samples.

**Western blot analysis**

Total protein of tissues or cells from different groups were extracted using RIPA buffer with protease inhibitor cocktail (complete mini-tablet, Roche, shanghai, China). The protein concentration was measured with a BCA protein determination kit (Solarbio, Beijing, China). Equal amounts of protein (40-60 μg) were separated by 10 % SDS-PAGE before being electro-transferred to a PVDF membrane (Millipore, Billerica, MA). After blocking with 5 % milk powder in TBS for 2 hours at room temperature, the membranes were incubated with specific anti-SYT1 (1:1000, ab131551, Abcam, Cambridge, UK), anti-JNK (1:1000, 9252, CST, Boston, USA), anti-p-JNK (1:200, 4668, CST, Boston, USA) or anti-P53 (1:1000, ab26, Abcam, Cambridge, UK) antibodies at 4 ˚C overnight. β-Actin (1:1000, 60008-1, Proteintech, Rosemont, USA) was used as internal references for total protein determination. The blots were visualized using an Odyssey infrared imaging system (LI-COR Biosciences, Lincoln, NE, USA), and the band intensities were quantified with the Image-Pro Plus 6.0 (Media Cybernetic, Silver and analyzed using the LI-COR Odyssey® scanner and software (LI-COR Biosciences).

**Vector construction and small RNA synthesis**

For over expression experiments, pEZ-p53 over expression vectors were purchased from GeneCoopoeia (Guangzhou, China). For shRNA-mediated knock-down experiments, pGpU6/GFP/Neo-P53 shRNA and negative control shRNA plasmid were purchased from GenePharma (Shanghai, China). Using genomic DNA from 293A cells as DNA templates, the promoter region of miR-34c ranging from -1425 bp to -790 bp (carrying the predicted P53RE), and from -2991 bp to -2683 bp (carrying the predicted P53RE) were amplified by PCR and inserted to pGL3 Basic vector (#cat E1751; from Promega, Madison, WI, USA) with NheI and HindIII enzymes, named as miR-34c-promoter-site1-Luc (PS1-WT) and miR-34c-promoter-site2-Luc (PS2-WT), separately. Then, their mutants were constructed using the QuickChange site-directed Gene Mutagenesis Kit (Stratagene, La Jolla, CA, USA). The luciferase vectors including 3′-UTR of syt1 mRNA (WT-psiCHECK-syt1-UTR (vector WT) or MT-psiCHECK-syt1-UTR-vector (vector MT) were purchased from NorClone Biotech (shanghai, China). Mus syt1 CDS was abtained by RT-PCR and inserted to GFP-N1 vector. syt1-siRNAs were purchased from NorClone Biotech (shanghai, China). miRNA mimics, miRNA inhibitors, miR-34c-neutralizing antagomir, and their controls were all designed and synthesized by RiboBio (Ribo Bio, Guangzhou, China). Attractene Transfection Reagent (Qiagen, Germany) was used for transfection experiments.

**Immunofluorescence staining**

Immunofluorescence staining analysis of mouse brain slices was performed by standard procedures. Tissues were fixed immediately in 3.7% paraformaldehyde (PFA), and postnatal mice were first deeply anesthetized and perfused with 3.7% PFA. The brains were isolated and fixed in 3.7% PFA at 4°C overnight and then transferred into 0.1 mol/L phosphate buffer containing 20-30% sucrose. Subsequently, serial 20-µm-thick sections were cut for immunofluorescence analysis. Each section was permeabilized with 1% Triton X-100 in Tris-buffered saline with Tween-20. After blocking with 10% goat serum (Zhongshan, China.) at room temperature for 2 h, the samples were incubated with anti-SYT1 (ab131551, Abcam), anti-P53 (ab26, Abcam), anti-microtubule Associated Protein-2 (MAP-2) (Bioworld Technology, Beijing, China) or anti-GFAP (Bioworld Technology, Beijing, China) at 4˚C overnight. Rabbit or mouse IgG was used as a negative control. After rinsing in 0.01 mol/L PBS, the sections were incubated with Cy3-conjugated goat anti-rabbit or mouse IgG (CWBio, Beijing, China) in the dark for 1 h at room temperature. The sections were rinsed with PBS and counter-stained with DAPI. The fluorescence intensity was measured in a fluorescence microscope or a confocal laser scanning microscope (Nikon, Tokyo, Japan). Immunofluorescence analysis was repeated at least 3 times at each stage with more than 3 independent mice.

**Analysis of dendritic spine density**

Primary hippocampal neurons were cultured as previously described. After 7 days in culture, AM34c or scrambled antagomir (50 nM) were added to the culture medium. After 48 h, cells were treated with 5 μM Aβ oligomer or scrambled Aβ oligomer. After additional 7 days, the neurons were stained with rhodamine-conjugated phalloidin (Invitrogen, Carlsbad, CA, USA). Under a fluorescence microscope, the spines were counted in 100 randomly selected dendritic segments from 30 cells in each group. To generate Aβ oligomer, 5 μM Aβ42 peptide or scrambled Aβ peptide (Millipore, Billerica, MA, USA) in dimethylsulfoxide was diluted to 100 μM with ice-cold culture medium, sonicated for 10 min, and incubated for 24 h at 4 ˚C.

**Golgi staining**

Golgi staining were performed following a protocols of FD Rapid GolgiStain™ Kit (FD, Columbia, USA).

**Novel object recognition test**

On day 1 of testing, mice were acclimated to the behavioral arena (40 cm × 40 cm × 30 cm tall white plastic box) for 5 min. For training session, after a 24 h period of acclimation, mice were placed into the center of the arena with two similar objects and allowed to explore the objects for 30 total visits but no longer than 10 min. The two identical objects were made of weighted plastic to prevent movement and located in the southeast and northwest quadrant, spaced equidistant from the arena walls. This training trial was completed twice, separated by a 10 min interval. Twenty-four hours following the last training trial, one object was replaced with a novel object; the object replaced was alternated for each mouse to avoid a side preference, and mice were again placed into the arena and allowed to explore the objects for 30 total visits or 10 min. An exploratory visit was defined as the mouse sniffing, climbing on, or touching an object or within 1 cm while facing an object. Testing was recorded with automated tracking system (AnyMaze; Stoelting Co.) and the arena was cleaned with 70% alcohol between trials to eliminate olfactory cues. An increased percentage of visits exploring the novel object (number of novel object visits/total visits × 100) was considered an index for improved long term memory retention in this task.

**Subjects and serum samples**

The patients with aMCI were diagnosed based on Petersen’s criteria: (1) memory complaint by participant or family; (2) normal activities of daily living; (3) normal general cognitive function; (4) objective impairment in one area of cognitive function as evidenced by scores of >1.5 SD of age-appropriate norms or abnormal memory function for age; (5) not demented. Well-trained research neurologists performed the diagnosis of patients with aMCI. For all subjects, Mini-Mental State Examination (MMSE) and Montreal Cognitive Assessment (MoCA) were also administered to determine cognitive status. Elders with severe psychiatric disorders, poor hearing and vision, and nervous system diseases were already excluded from both cases and controls by investigating past medical history. Fasting blood samples (5 ml) were collected in BD Vacutainer SST tubes (BD, New Jersey, NJ, EEUU) from each subject within a few days after psychological testing. Tubes were kept in vertical position for 30 min to allow clot formation and centrifuged at 3,000 rpm for 10 min at room temperature (15-25˚C), followed by centrifugation at 16,000 rpm for 10 min in 4˚C The serum was collected gently and stored at −80˚C for further analysis. Repeated freeze/thaw cycles were avoided.

**Supplementary results**

Figure S1


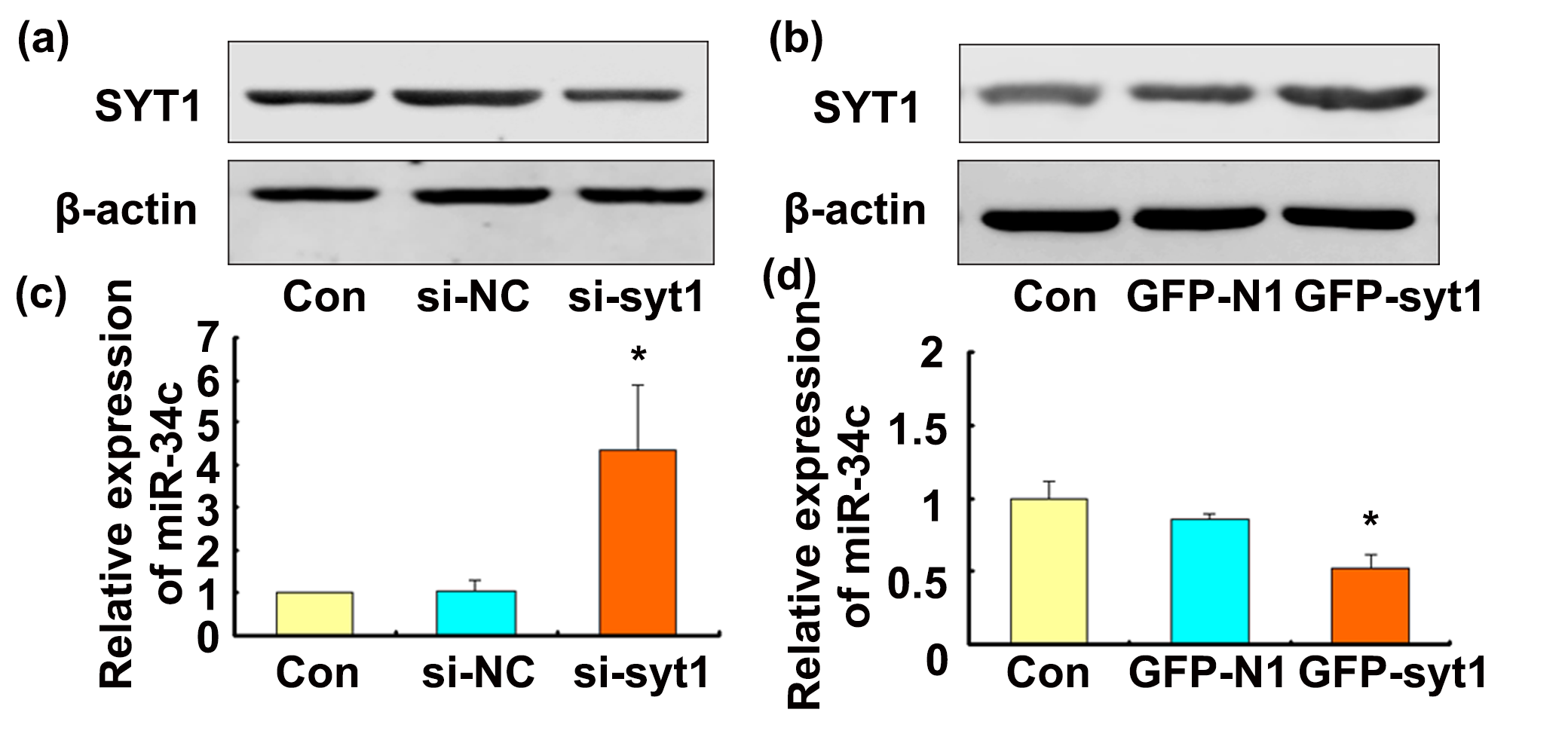


Figure S1 Expression levels of miR-34c in HT-22 cells transfected with GFP-syt1 plasmid or si-syt1. **P* < 0.05, *vs* si-NC or GFP-N1. Each bar represents the mean ± SD of three independent experiments.

Table S1

Demographics of the study subjects

|  | NC  (n = 69) | aMCI  (n = 71) | *P* |
| --- | --- | --- | --- |
| Age（years）  (mean ± SD) | 74.17 ± 6.87 | 73.89 ± 6.43 | 0.386 ^a^ |
| Sex  (male: female) | 27:42 | 32:39 | 0.249 ^a^ |
| MMSE | 27.62 ± 2.12 | 21 ± 4.41 | <0.0001 ^a^ |
| MoCA | 24.7 ± 3.71 | 17.45 ± 5.06 | 0.0085 ^a^ |
| miR-34c | -4.10 ± 1.50 ^b^ | -4.54 ± 2.30 ^b^ | 0.034 ^c^ |

Note: *a,* Compared by t-test between aMCI and NC groups. b, Values were expressed as median of lg2 ^-△Ct^. c, Compared by Mann-Whitney U-test between aMCI and NC groups.
